# Predicting Chronic Subdural Hematoma Recurrence and Stroke Outcomes While Withholding Antiplatelet and Anticoagulant Agents

**DOI:** 10.3389/fneur.2019.01401

**Published:** 2020-01-15

**Authors:** Mario Zanaty, Brian J. Park, Scott C. Seaman, William E. Cliffton, Timothy Woodiwiss, Anthony Piscopo, Matthew A. Howard, Kingsley Abode-Iyamah

**Affiliations:** ^1^Department of Neurosurgery, University of Iowa, Iowa City, IA, United States; ^2^Department of Neurosurgery, Mayo Clinic, Jacksonville, FL, United States

**Keywords:** chronic subdural hematoma, recurrence, stroke, antiplatelet, anticoagulation, oral anticoagulation, machine learning

## Abstract

**Introduction:** The aging of the western population and the increased use of oral anticoagulation (OAC) and antiplatelet drugs (APD) will result in a clinical dilemma on how to balance the recurrence risk of chronic subdural hematoma (cSDH) with the risk of withholding blood thinners.

**Objective:** To identify features that predicts recurrence, thromboembolism (TEE), hospital stay and mortality. To identify the optimal window for resuming APD or OAC.

**Methods:** We performed a retrospective multivariate analysis of a prospectively collected database. We then build machine learning models for outcomes prediction.

**Results:** We identified 596 patients. The rate of recurrence was 22.17%, that of thromboembolism was 0.9% and that of mortality was 14.78%. Smoking, platelet dysfunction, CKD, and alcohol use were independent predictors of higher recurrence, while resolution of the SDH was protective. OAC use had higher odds of developing TEEs. CKD, developing a new neurological deficit or a TEEs were independent predictors of higher mortality. We find the optimal time of resuming OAC to be after 2 days but before 21 days as these patients had the lowest recurrence of bleeding associated with a low risk of stroke. The ML model achieved an accuracy of 93, precision of 0.84 and recall of 0.80 for recurrence prediction. ML models for hospital stay performed poorly (*R*^2^ = 0.33). ML model for stroke was overfitted given the low number of events.

**Conclusion:** ML modeling is feasible. However, large well-designed prospective multicenter studies are needed for accurate ML so that clinicians can balance the risks of recurrence with the risk of TEEs, especially for high-risk anticoagulated patients.

## Introduction

The aging of the western population and the increasing use of oral anticoagulation (OAC) and antiplatelet drugs (APD) will likely result in an increased incidence of chronic subdural hematoma (cSDH) (1). Elderly patients have a 10-fold increase in the risk of developing cSDH, estimated around 58–200 per 100,000 per year, depending on their anticoagulation status (2). Despite technological advances in the neurosurgical field, there has little improvement regarding the surgical technique for cSDH, an entity that has been well-characterized since 1857 (3) and treated with trepanation since prehistoric ages (4).

Although the commonly used techniques are relatively safe and simple (1), cSDH have a mortality rate as high as 42% (5, 6) and a recurrence rate up to 76% (1, 7, 8). Prediction models so far have been inconsistent (9–12) and are not routinely implemented in clinical practice. In addition, questions regarding postoperative OAC and APD management remain unanswered (1).

We aimed to identify features that can predict cSDH recurrence, thromboembolic events (TEE), hospital stay and mortality, using biostatistical analysis and machine learning (ML) models. We then aimed to assess the optimal timing for resuming oral anticoagulation drugs (OADs) or antiplatelet drugs (APDs) by balancing the risk of recurrence with that of thromboembolic events.

## Methods

### Patient Characteristics

We performed a retrospective review of patients with cSDH from a single institution between 2007 and 2015 after Institutional Review Board approval. We identified 596 patients, 505 of which had unilateral cSDH (84.7%). Inclusion criteria were age > 40 years and cSDH requiring evacuation by burr-hole drainage. We collected data on age, hypertension, diabetes mellitus status, body mass index (BMI), chronic kidney disease (CKD) (defined as > stage 3), smoking (> 1 pack per day), chronic alcoholism (>3 glasses a day), platelet disorder (pancytopenia), OAC use, aspirin use and dosage, clopidogrel use, history of cerebrovascular accident, history of atrial fibrillation, history of mechanical valve, and history of liver disease. The size of the subdural hematoma on preoperative and postoperative scan was recorded as follows: maximum height on the coronal scan, maximal length on the axial scan and maximal width or thickness on the axial scan. The cortical atrophy grade was measured using Brickman et al. protocol (13), as it has been shown to correlate with a faster decline in cognition in Alzheimer's disease (13). We measured the largest distance between the heads of the caudate and divided it by the width of the skull (from inner table to inner table) on the same slice. Thus, enlarged ventricles increase the value and indicates more atrophy. For time off OAC, patients were classified into the following categories: (1) resumption within the first 2 days post-op, (2) resumption between day 3- and 2-weeks post-op, (3) resumption between 2- and 3-weeks post-op, (4) resumption between 3- and 4-weeks postop, and (5) resumption between 4- and 6-weeks post-op. The same subcategorization was performed for patients on APD. All patients were treated with burr-hole trephination with a closed drainage system. They remained bed rest for 12–24 h post-op and a CT scan was obtained prior to removal of the drain and slow mobilization. During the first 24 h patients were given intravenous fluids unless contra-indicated. Subcutaneous heparin was started after 48 h on all patients who required help with ambulation.

### Outcome Assessment

Recurrence was defined as the accumulation of chronic subdural fluid requiring reoperation after at least 48 h from the initial surgery and after discharge. We have chosen 48 h as a cut-off since a postop CT scan is obtained within 24 h. If the surgeon did not properly evacuate the SDH, the patient is taken back to the OR for a re-do surgery during the same hospital stay. Thus, a second surgery performed during the same hospitalization was not considered as a recurrence. Thromboembolic events (TEEs) were defined as transient ischemic attacks or strokes during the follow-up period after surgery. Duration of hospitalization was recorded in days. The size of the postoperative cSDH was measured by the same method as the preoperative cSDH size. The rate of developing post-op neurological deficits and mortality was recorded. New neurological deficit was defined as a neurological deficit that was not present preop. This could be due to seizures, postop bleeding, or stroke (from stopping the anticoagulation or antiplatelets). Death from a neurological injury was defined as death as a result of postoperative stroke, hemorrhage or seizures (which includes withdrawal of care and death from the reasons above). The risk of developing a stroke or a cSDH recurrence in relation to the timing of recommencement of APD or OAC was assessed. Complete resolution of the SDH was defined as the complete disappearance of the cSDH on CT scan on the last follow-up. The mortality was defined as the rate of death from hospitalization to follow-up.

### Surgical Method

Patients underwent endotracheal intubation with general anesthesia. For patients who underwent a single burr hole placement, the burr hole was placed at a point that intersects the superior temporal line with a vertical line from the tip of the mastoid (Euryon), which is the area of maximal atrophy. For patients who underwent 2 burr hole placement, a burr hole was placed in the frontal area and one in the parietal area. The exact location was estimated with the help of the CT scan preop. All burr holes were placed with the craniotome. A subgaleal drain (passive system) was always used (it was tunneled from the posterior burr hole when 2 burr holes were placed). The dura was cauterized and opened. Suction irrigation was used to retrieve the clot, and the membrane was open when present. When 2 burr holes were placed, we ensured that irrigating the frontal one lead to drainage of the irrigation solution from the posterior one.

Hemostasis and closure were performed in the usual setting. Of note, a red rubber catheter was used depending on the surgeon's preference. Hemostasis and closure were performed in the standard fashion. The patient remained flat for 24 h duration post surgery. The subgaleal drain was removed at the surgeon's discretion, typically 24 h post surgery.

### Statistical Analysis

Patients with unavailable data were excluded from the analysis (<1%). Categorical data were transformed to dummy variables. Descriptive statistics was first performed followed by data visualization. Univariate analysis was carried out to test for significance (Table 1). Features with *p* < 0.20 were included in the multivariate analysis. A logistic regression model was performed for categorical targets and a multivariate linear regression model was used for numerical targets. A multivariate linear regression model was performed when the data was linear and there was no heteroscedasticity (if not a log transformation or scaling was performed). We tested for autocorrelation through the Durbin-Watson test (accepted values between 1 and 3) and for multicollinearity through the variance inflation factor and excluded features with a factor > 6. Analysis was performed using Python 3.7 in Spyder 3.6 (Anaconda distribution). Stats model library was used for univariate and multivariate analysis.

**Table 1:** Demographics and characteristics of the study population.

**Features**	**Descriptions**
Age	73 ± 13
Unilateral cSDH	84.73%
Male	66.67%
BMI	26.84 ± 5.8
Hypertension	53.68%
Diabetes	24.03%
Chronic Kidney disease	7.56%
Ventriculoperitoneal shunt	4.84%
History of stroke/T.I.As	12.79%
Platelet dysfunction	3.48%
ASA 81	29.45%
ASA 325	15.69%
Clopidogrel	10.07%
Warfarin	20.15%
Smoking	37.21%
Chronic alcohol disease	33.72%
Liver disease	2.91%
**Preop**	
Height	9.11 ± 1.60 mm
Width	1.98 ± 0.93 mm
Length	12.15 ± 2.22 mm
Cortical atrophy grade	0.16 ± 0.05

*Values reported as mean (SD) when appropriate*.

### Machine Learning Modeling

Machine learning modeling was performed to predict hospital stay, recurrence, and stroke risk (see Supplementals 1–3 for details). We excluded patients with unavailable data from the machine learning model. Supplemental 1 displays our model pipeline. We then standardized all numerical features. For all machine learning models, the features included in the analysis were those with significant F value on the univariate analysis (Scikit learn using F_Classif when the target is categorical and F_regression for numerical target). The target value was defined as the actual value of the independent variable. The predicted value was defined as the predicted value of the independent variable. For numerical values, the residual is defined as the target minus the predicted values. The data was split randomly into 80% for training + validating and 20% for testing, with stratification. A gridsearch with 10-fold cross validation for every model was performed to establish the best hyperparameter and the best score for each model, based on the training/validation data only. The best scores for classifiers were the accuracy score, F1 score, recall and precision. The best model was then chosen and tested on the testing dataset. For regression models, we assessed the linearity by grid search to obtain the best kernel for the SVC model. However, models for both linear and non-linear data were tested and evaluated based on the parameters above. Analysis was performed using Python 3.7 in Spyder 3.6 (Anaconda distribution) using SciKit learn for ML and Yellow brick for data visualization and ML.

## Results

### Patients Demographics and Outcomes

We identified 596 patients and excluded 10 patients from the inferential analysis and 30 patients from the ML models due to unavailable data on certain features. Patients demographics and characteristics are listed in Table 1. The average age was 73 ± 13 years with a range of 40–97 years. Males constituted 66.67% of the population. Chronic kidney disease affected 7% of patients. Twenty percent used OAC (98.83% of which were on warfarin) and 46% used APD. The average BMI was 26.8 ± 5.8. Burr-hole trephination significantly reduced the SDH height by 2 mm (*p* < 0.001) and length by 4 mm (*p* < 0.001) (Tables 1, 2). It did not affect the thickness. The majority of the patients (84.73%) had unilateral cSDH. New neurological deficit occurred in 6.8% of the postoperative patients and included: seizures, stroke, worsening of previous symptoms or post-op hemorrhage. Liver disease affected 1.9% (2.9) of the population and these patients were excluded from the inferential analysis.

**Table 2:** Outcomes.

**Features**	**Descriptions**
**Postop**	
Height	7.32 ±1.98 mm
Width/Thickness	1.13 ±0.50 mm
Length	8.67 ±2.85 mm
SDH resolution	54.47%
Rebleed	22.17%
TEEs	0.90%
New neurological deficit	6.8%
Death	14.7%

### Predictors of SDH Recurrence

The rate of recurrence was 22.17%. For patients on blood thinners, the risk of recurrence was significantly higher, and that of patients with CKD and platelet dysfunction was the highest (Table 3). Univariate analysis showed CKD, OAC use, diabetes, smoking, alcohol, platelet dysfunction, having a shunt, pre-operative height, and post-operative height of the SDH were associated with higher odds of recurrence. We found that patients with CKD and those with platelet dysfunction have 2–3 times (platelet disorder OR = 2.74, CKD 2.332) the odds of developing a recurrent bleed on multivariate analysis (Table 3). Resolution of the SDH was protective. On multivariate analysis, smoking, platelet dysfunction, CKD, and alcohol use were independent predictors of higher recurrence, while resolution of the SDH remained protective. The number of burr-holes used to treat each cSDH did not affect the outcome, although 2 burr-holes per side were used in 78.5% of the cases (468 patients).

**Table 3:** Predictors of recurrence.

	**Odds ratio**	***P***
**Univariate analysis**		
SDH resolution	0.627	0.029^*^
Chronic Kidney Disease	2.690	0.004^*^
Diabetes	1.13	0.159^*^
Smoking	1.692	0.104
Alcohol	1.623	0.059
OAC	1.335	0.018^*^
Preop Height	1.174	0.032^*^
Postop Height	1.145	0.091
Clopidogrel	1.23	0.03^*^
Shunt	2.25	0.004^*^
Platelet disorders	1.76	0.005^*^
**Multivariate analysis**		
Smoking	4.867	0.001^*^
SDH resolution	0.271	0.001^*^
Platelet disorder	2.74	0.030^*^
Chronic Kidney Disease	2.332	0.020^*^

*Predictors with p < 0.20 were included in the multivariate analysis*.

*Predictors with a p < 0.20 on univariate and <0.05 on multivariate analysis were reported*.

* *Statistically significant*.

### Predictors of Thromboembolic Events

A small number of patients experienced a TEE (0.9%;6/596), all of which were deemed embolic. Univariate analysis showed OAC use, diabetes and a longer time off anticoagulation were associated with higher odds of developing TEEs (Table 4). On multivariate analysis, only OAC use had higher odds of developing TEEs, although the low number of TEEs limits this analysis.

**Table 4:** Predictors of TEEs.

	**Odds ratio**	***P***
**Univariate analysis**		
OAC	6.030	0.051^*^
Diabetes	4.835	0.086
Time off oral anticoagulation	0.5	0.182
**Multivariate analysis**		
OAC	3.275	0.049^*^

*Predictors with p < 0.20 were included in the multivariate analysis*.

*Predictors with a p < 0.20 on univariate and <0.05 on multivariate analysis were reported*.

* *Statistically significant*.

### Predictors of Mortality

The overall mortality rate was 14.78%. Causes of mortality were the following: neurological injury (6.80%), kidney failure (0.60%), and other causes (10.10%), such as liver disease, cardiac failure, and unknown cause of death in the community. Neurological injury included postoperative complications such as postoperative acute SDH and refractory seizures, ischemic stroke during hospital stay or at follow-up, and development of an acute SDH in a delayed fashion (after discharge) due to blood thinners, fall, or unknown reasons. Direct postoperative complications (acute SDH, refractory seizures) accounted for 2.01% (12 cases) of the population. Univariate analysis showed CKD, diabetes, recurrence of the SDH, developing a TEE or developing a new neurological deficit were associated with higher mortality, while resolution of the SDH was protective (Table 5). On multivariate analysis, CKD, developing a new neurological deficit or a TEEs were independent predictors of higher mortality.

**Table 5:** Predictors of mortality.

	**Odds ratio**	***P***
**Univariate analysis**		
SDH resolution	0.262	0.001^*^
Smoking	1.95	0.068
Platelet disorder	1.17	0.029^*^
CKD	2.868	0.005^*^
DM	1.688	0.052
New neurological deficit	6.755	0.001^*^
Rebleed	1.795	0.033^*^
Thrombotic event	9.000	0.017^*^
Warfarin	1.04	0.20
**Multivariate analysis**		
New neurological deficit	6.051	0.001^*^
SDH resolution	0.306	0.001^*^
Thrombotic event	9.723	0.025^*^
CKD	2.705	0.019^*^

*Predictors with p < 0.20 were included in the multivariate analysis*.

*Predictors with a p < 0.20 on univariate and <0.05 on multivariate analysis were reported*.

* *Statistically significant*.

### Predictors of Duration of Hospitalization

Most frequent hospital length was 3 days. The median and mean were 4 and 6 days respectively. This was due to the range of from 2 to 57 days, making the distribution heavily skewed. Univariate analysis showed use of clopidogrel, higher BMI, and larger SDH were associated with a longer length of stay, while SDH resolution was associated with a shorter hospitalization (Table 6). On multivariate analysis, the combined use of OAC and APD was significantly associated with a longer hospitalization.

**Table 6:** Predictors of longer hospital stay (predictors that increase the length of stay).

	**Odds ratio**	***P***
**Univariate analysis**		
Alcohol	1.6	0.073
History of cerebrovascular event	3.678	0.072
Clopidogrel	2.2	0.128
SDH resolution	0.515	0.193
Male	2.36	0.109
Preop average size	2.121	0.035^*^
Preop height	3.90	0.003^*^
Preop length	1.96	0.076
Postop height	1.392	0.065
BMI	4.835	0.173
**Multivariate analysis**		
OAC+APD	3.275	0.049^*^

*Predictors with p < 0.20 were included in the multivariate analysis*.

*Predictors with a p < 0.20 on univariate and <0.05 on multivariate analysis were reported*.

* *Statistically significant*.

### Optimal Window for Resuming Blood Thinners

**Figure 1:**
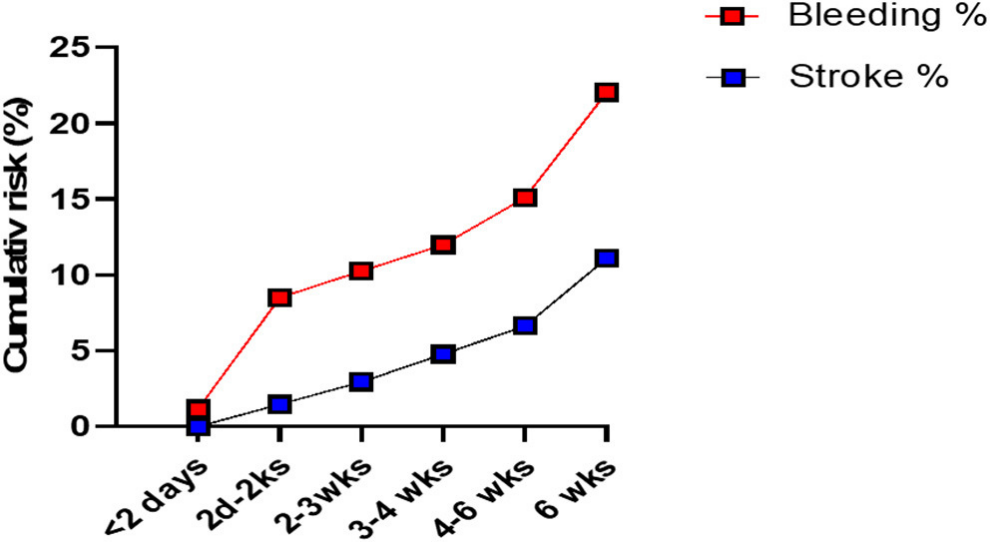
The cumulative risk of recurrence of the chronic SDH (“Bleeding”) and that of stroke in patients with cSDH (all patients combined). The cumulative risk of recurrence increases with time. There are 2 periods of higher risk (dramatic increase in the curve) at 2 days to 2 weeks post-op and after 6 weeks post-op. The risk of stroke gradually increases with time.

**Figure 2:**
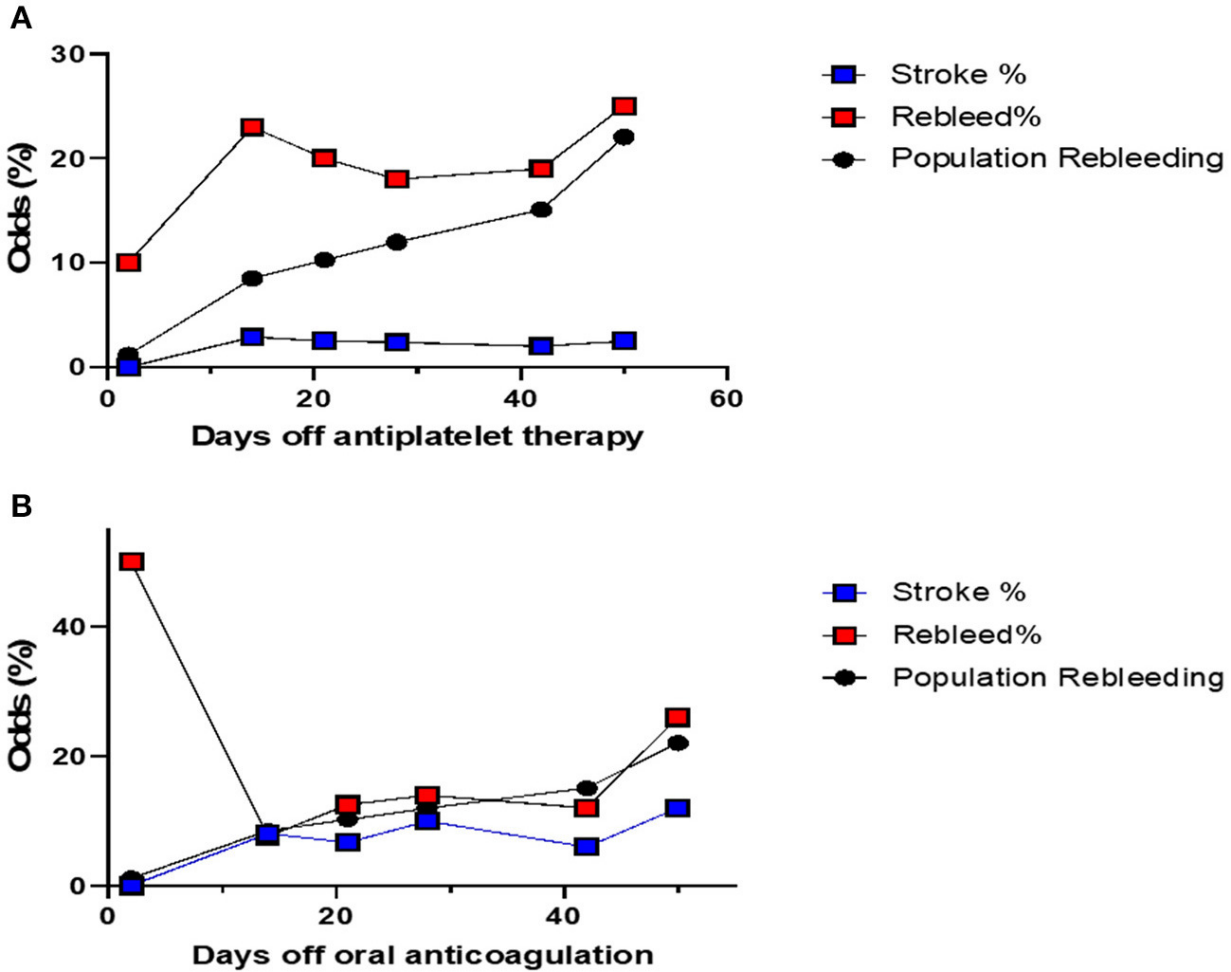
**(A)** The odds of stroke and recurrence in patients with cSDH on APD, compared with the risk of recurrence in patients not on any blood thinners. This risk is calculated separately for each cohort of patients depending on when their APD were resumed. The risk of recurrence is always higher depending on the duration of withholding therapy. Patients where the APD was resumed between day 2 and week 2 post-op, had the highest risk, although that risk is not significantly different from the rest. Patients who resumed their APD within 48 h had lower odds of recurrence, but this is limited by the small sample size. The odds of stroke are similar. **(B)** The odds of stroke and recurrence in patients with cSDH on OAC, compared with the risk of recurrence in patients not on any blood thinners. This risk is calculated separately for each cohort of patients depending on when their OAC were resumed. The risk of recurrence is highest for patients who resumed their OAC within the first 48 h. The risk of stroke increases with time. The optimal time to restart OAC would be between 2 days and 20 days post-op.

The cumulative risk of recurrence of cSDH appears to increase with time (all patients combined) (Figure 1). The risk of stroke appears also to gradually increase with time. The chance of developing a recurrence was highest when APD was resumed 2–14 days post-op (Figure 2A). However, this rate is not clinically different from other duration of holding therapy. The low chance of developing a recurrence in patients where the APD was resumed prior to 48 h is biased by the small sample size. The chance of developing a recurrence was highest for OAC when resumed within the first 48 h (Figure 2B). It then decreases quickly and stabilizes if the OAC were resumed after 2 days. Thus, the lowest risk of recurrence was the resumption of OAC between 2 and 20 days, with a slight increase after 20 days. The chance of developing TEEs is small and exhibits a small increase with longer time off APD or OAC. We find the optimal time of resuming OAC to be after 2 days but before 21 days as these patients had the lowest recurrence of bleeding associated with a low risk of stroke (Figure 2). We did not find the best duration to resume therapy as the risk was always higher than the general population without a specific peak. However, the data suggests a reasonably good safety prole of holding anticoagulation up to day 42.

### Machine Learning Models

The optimal classifier to predict recurrence was the RFT with a f1-score of 0.92 and an area under the curve (AUC) of 0.91 based on the 10-fold cross validation using grid search. This model was then tested on the test data and achieved an accuracy of 93%, precision of 0.84 (specificity = 84%) and recall of 0.80 (sensitivity = 80%) for predicting recurrence (Supplemental 1). We could not perform an ML model to predict TEE due to the lack of TEE events (0.09%). Finally, we tested an ML model for the hospital stay. The multivariate linear regression using ML technique had an *R*^2^ = 0.15. The RFT model had an *R*^2^ = 0.33 outperforming all other models, albeit still having a poor prediction accuracy, as displayed in the residual plots and the difference between predicted and target values plot (Supplementals 2, 3). Thus, the variation in the selected features explain only 33% of the variation in the hospital stay, making our model not useful. This is not surprising giving the heavy skewed distribution in the hospital stay, along with other features that could affect the prediction but were not included in the model (insurance, availability of the rehabilitation facility, etc.). Thus, a ML model for hospital stay may be feasible, but it should be a separate study, and must include insurance and other factors, which will vary according to state, hospital policy, and setting (private vs. academic).

## Discussion

Resolution of cSDH is protective against recurrence and is associated with shorter hospitalization, while APD, OAC, CKD, platelet disorders, and alcohol had a higher rate of recurrence. Meanwhile, OAC had a higher risk of stroke likely due to the risks associated with pausing the anticoagulation. CKD patients present complex management issues, as this patient population often have baseline platelet dysfunction due to uremia and often cannot be resuscitated postoperatively with high volume of fluids to help brain expansion. Burr-hole trephination reduced the height and length but did not affect the thickness/width, which is likely related to the brain volume. We have found, similar to Stanišic et al. (12), that large pre- and post-operative SDH volume tend to have a higher recurrence rate, whether the lesion is bilateral or not. Stanišic et al. (12) also showed that bilateral cSDH is not predictive of postoperative recurrence, nor is it a surrogate for the cSDH volume, as the preoperative and postoperative volume of a unilateral CSDH in one patient can have a larger volume than bilateral SDH in a separate patient (14). Similarly, Xu et al. (15) showed that bilateral cSDH were not associated with recurrence; however, with 44 bilateral cases and 10 unilateral cases, the number of patients in that study was small. In contrast, Shen et al. (16), reviewed 102 patients where bilateral cSDH was an independent risk factor for recurrence. The controversy regarding the role of bilateral cSDH in the rate of recurrence may be confounded by the cSDH volume or brain atrophy. We did not find brain atrophy to be related to recurrence unlike Shen et al. (16), but the measurement techniques were different. OACs increase the risk of cSDH 4- to 15-fold (17) depending on the intensity of anticoagulation, (17) patient's age (18); cerebral atrophy may also increase this risk. We did not find age or cortical atrophy grade to be independent predictors for recurrence, although the combined interaction of cortical atrophy with OAC was a predictor of recurrence Similarly, a 5-year retrospective study of 248 patients showed that anticoagulation in addition to headache and preoperative midline shift was an independent predictor for recurrence (9). In that study however, the risk of recurrence was lower than ours (12.6% vs. 22.17%), which may be due to the duration of follow-up.

Interestingly, the risk of recurrence is high when the OAC or APD are started within 48 h post-op. We have also shown that the risk of recurrence increases with time for the first few months. This is likely due to the slow reaccumulation and the time it takes to become symptomatic. Patients that started their OAC between 2 days and 2 weeks post-op had lower rate of rebleeding compared to those who were started within the first 48 h and similar rates to those started after 2 weeks. This is not consistent with a retrospective study of 187 patients where postoperative warfarin resumption within 2–3 days did not affect the recurrence rate (19). In our study, the cohort that resumed the APD or OAC after 2 days post-op, but before 2 weeks, had the same risk of stroke compared to those who resumed their blood thinners within the first 48 h and less than those who did after 2 weeks. The low rate of TEE, however, is a limiting factor for such analysis. In addition, a TEE is associated with a higher mortality on multivariate analysis, while a recurrence by itself is not. In addition, TEEs may result in a poor quality of life compared with cSDH recurrence. This could explain the practice of some neurosurgeons who immediately resume OAC and APD, especially in the setting of a fresh stent or a prosthetic valve, fearing a devastating TEE. Given the results of this study, perhaps patients with high risk of bleeding and low risk of TEEs can resume the anticoagulation at the end of the 2-weeks post-op period, while those with high risk of stroke and low risk of bleeding can resume their blood thinners after 2 days post-op to optimize the balance between risk of recurrence and TEE. Patients with high risk of bleeding and stroke can be followed more closely or referred to the neurosurgeon for routine CT scan to decide when to resume the blood thinner. It is foreseeable in the future that ML models with accurate predictions will help the discussion between neurologists, cardiologists, hematologist and neurosurgeons to balance the risks of stroke with that of bleeding.

The ML model for recurrence has performed well on the testing data. The 93% accuracy of the model however is misleading; a model that predict no recurrence in all cases will be 78% accurate, as the risk of bleeding is 22%. Thus, our accuracy should be at least >80 for the model to be practical. However, a recall or a precision of a model that predicts no bleeding at all times will be 0. One must carefully evaluate the precision and recall for both recurrence and non-recurrence, and for TEE and non-TEE predictions. The best model would be one that has a higher recall for stroke (minimal false negative and thus highly sensitive, i.e., “sensitivity”) and a higher precision for bleeding (minimal false positive and highly specific, i.e., specificity). ML model for recurrence was feasible while that for TEE was not due to the low frequency of these events. More data stratified by stroke risk features (such as prosthetic valve, atrial fibrillation, etc.) will be needed. The sensitivity and specificity of the model limits its use, and call for a multicenter collaboration in order to develop accurate models, available and easy to deploy in referral centers and in the community. Unsurprisingly, hospital stay was unpredictable as it is related to insurance, premorbid status, the availability of physical therapist and social worker, and the occupying state of the rehabilitation or skilled facility chosen by the patients. These data were not available for analysis.

## Limitations

Although there is no consensus on the definition of recurrence, we defined it as symptomatic reaccumulation requiring re-operation. Thus, patients with radiographic recurrence were analyzed with the no recurrence group. In addition, patients are usually followed at 4–6 weeks follow-up, unless their symptoms recur and they return to the hospital. At the follow-up period is the typical diagnosis of recurrence. This may be one confounding factor for having higher recurrence at 4–6 weeks, while in reality the accumulation would have started earlier. In addition, it is hard to tease out a “bad evacuation” requiring a delayed second surgery from a true recurrence, and this may bias the results. However, the period 4–6 weeks reflect practice in the community. In our study, warfarin was the most used OAC, while recently there has been a trend toward using novel agents. Thus, these results may not be generalizable for patients on novel OAC. Another consensus limitation is the definition of mortality in cSDH. We have found mortality to be related to patient's comorbidities, neurological injury, withdrawal of care and old age, making it difficult sometimes to differentiate cSDH-related mortality from other causes. Overall, the cause of mortality in this population is similar to that in the general elderly population. Thus, a ML prediction model for mortality in cSDH will not be useful. However, we have identified that a post-op neurological deficit and TEEs are independent predictors of mortality. For the reasons above, one must be careful before committing high-risk patients for operative management. We did not evaluate the functional disabilities of patients, particularly after reoperation or after experiencing TEEs. The main limitation with TEEs detection is the short observation period. Another important factor in risk decision making for restarting APD is that these commonly are prescribed for cardiac stents; as such information regarding NSTEMI/STEMI while off APD would be useful in this risk stratification process and thus is a limitation. We also did not include other features such as pre-albumin, or neurological status on admission, which could bias the outcome (6). Finally, the heterogeneity of studied predictors, management strategies, and comorbidities do not allow for a comparison between clinical studies (12). A reliable ML model will need more patients, from different centers (to avoid selection bias), in a collaborative prospective well-designed trial. However, the findings from this series are promising.

## Conclusion

Predictions in cSDH continue to represent a challenging problem. We highlighted features that predict recurrence, higher risk of TEEs and higher mortality. An optimal timing window for OAC is between 2 days and 21 days postop. While a useful ML model for recurrence was feasible, that for TEEs and duration of hospitalization were not. Large well-designed prospective multicenter studies are needed to build prediction models so that clinicians can balance the risks of recurrence with the risk of TEEs, especially for high-risk anticoagulated patients.

## Data Availability Statement

All datasets generated for this study are included in the article/Supplementary Material.

## Ethics Statement

The studies involving human participants were reviewed and approved by IRB University of Iowa. Written informed consent for participation was not required for this study in accordance with the national legislation and the institutional requirements.

## Author Contributions

KA-I and MZ contributed conception and design of the study. All authors contributed to data analysis, writing, revision, reading and approving final versions of the manuscript.

### Conflict of Interest

The authors declare that the research was conducted in the absence of any commercial or financial relationships that could be construed as a potential conflict of interest.
