# Supplementary material for: Predicting Chronic Subdural Hematoma Recurrence and Stroke Outcomes While Withholding Antiplatelet and Anticoagulant Agents
Source: Front Neurol. 2020 Jan 15;10:1401. doi: 10.3389/fneur.2019.01401 (PMC6974672; doi:10.3389/fneur.2019.01401)
Supplement: Supplementary file 1 [file Table_1.DOCX]

**SUPPLEMENTAL**


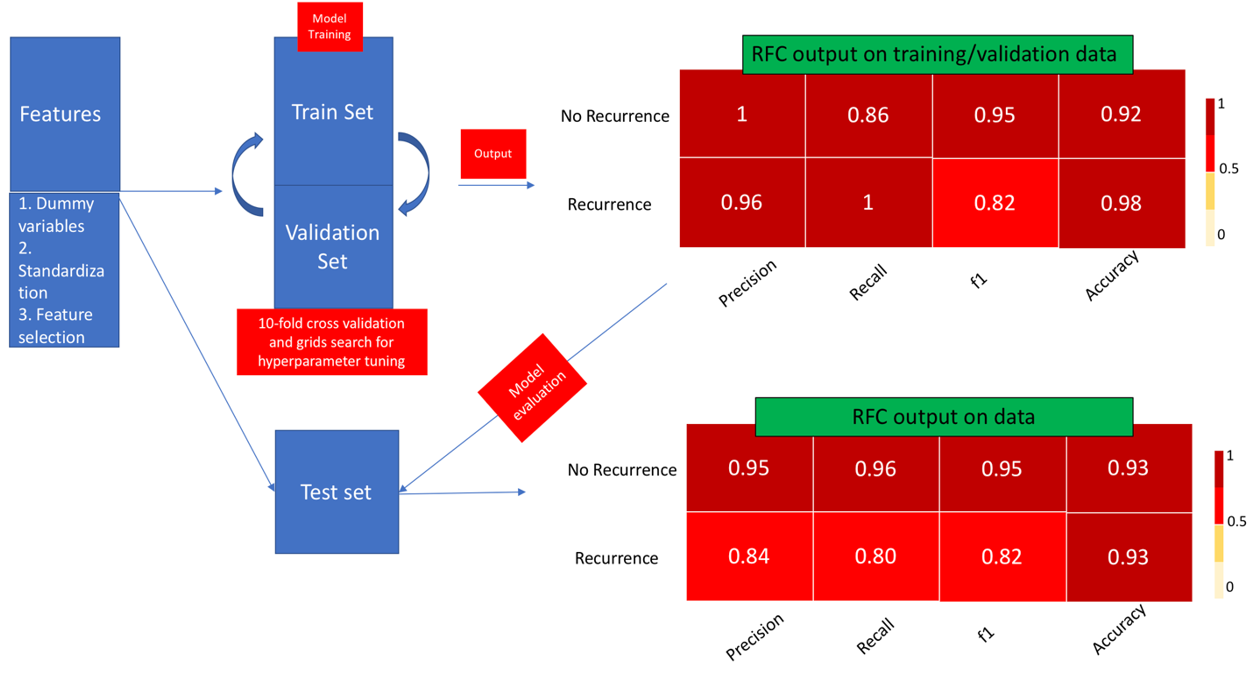


**Supplemental 1:** Model pipeline. We created dummy variables for the categorical features. We then standardized all numerical features. The data was then balanced for rebleed in a 60-40% split to keep a high number of patients and was then shuffled to remove time bias. The data was split randomly into 80% for training + validating and 20% for testing, with stratification. A gridsearch with 10-fold cross validation for every model was performed to establish the best hyperparameter and the best score for each model, based on the training/validation data only. The best scores for classifiers were the accuracy score, F1 score, recall and precision. The best model was then chosen and tested on the testing dataset. To choose the best classifier, we relied on its performance on the testing dataset by computing its precision, accuracy, recall and F1 score. We tested the following classifiers to predict the risk of rebleeding: stochastic gradient descent (SDG), extra tree classifier (ETC), random forest classifier (RFC), support vector machine classifier (SVC), logistic regression CV (LRCV), and logistic regression.


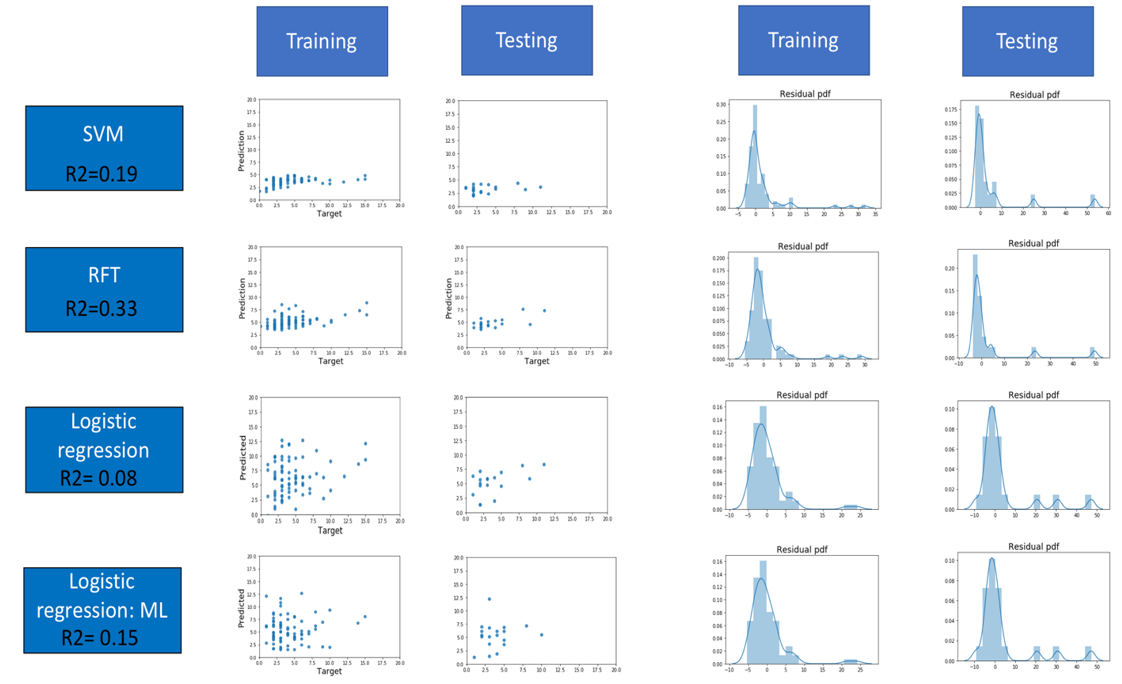


**Supplemental 2:** ML model for hospital stay:We tested the following regressors to predict hospital stay: RFT, support vector machine, and multivariate linear regression. W assessed the linearity by grid search to obtain the best kernel for the SVC model. However, models for both linear and nonlinear data were tested and evaluated based on the parameters above To choose the best regressor, we relied on performance on the testing dataset and R2 score, the percent difference between predicted and target values, the distribution of predicted and target values around the 45-degree line- in the predicted vs. target values scatter plot-, and on the shape and distribution of the residuals plot.


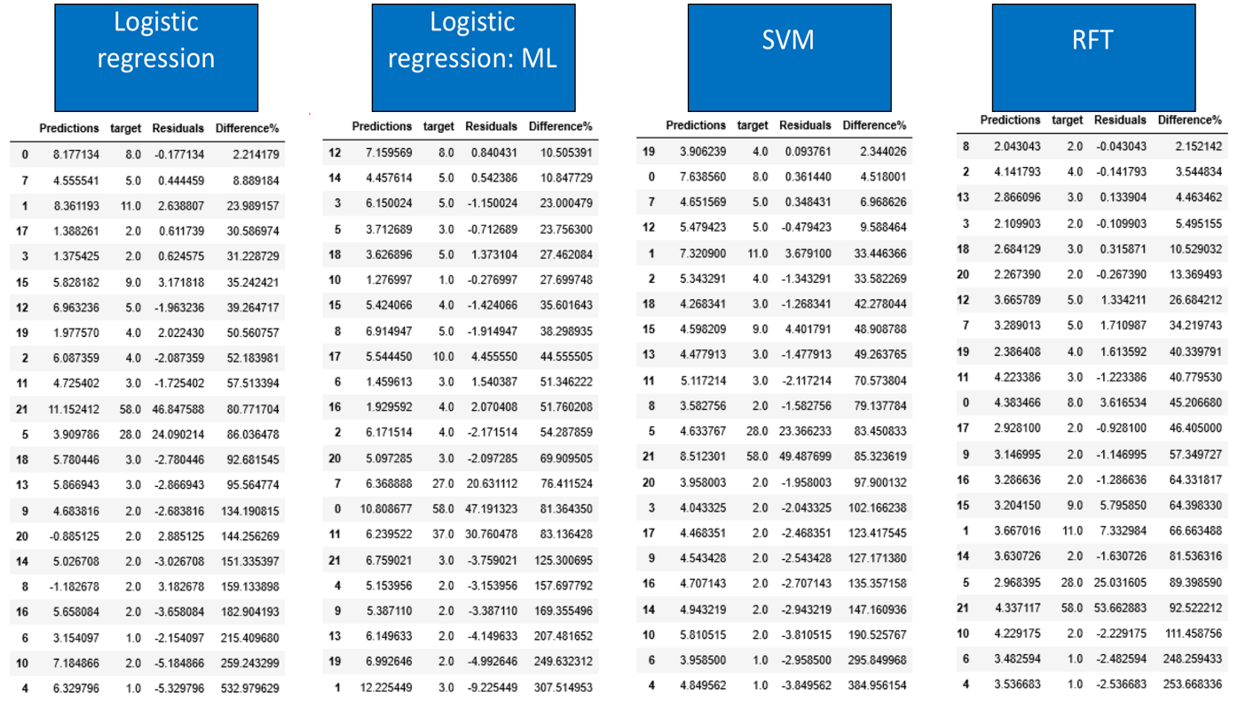


**Supplemental 3**: Predictions for hospital stay in days. The actual value, predicted value, residual (predicted-actual) and the difference (%) are displayed. These samples demonstrate the difficulty with predicting hospital stay in days fir patients with cSDH, due to many complicated factors (insurance, physical therapy availability, weekend and holidays, etc.)
